# Comprehensive analysis of the Caffeic acid O-methyltransferase gene family in kenaf (*Hibiscus cannabinus* L.) and their expression characteristics in response to salinity stress

**DOI:** 10.3389/fpls.2025.1678383

**Published:** 2025-10-07

**Authors:** Jiantang Xu, Tianjin Liu, Hui Lin, Rong Huang, Meixia Chen, Pingping Fang, Xiaoping Niu

**Affiliations:** ^1^ Key Lab of Genetics, Breeding and Multiple Utilization of Crops, Ministry of Education, College of Life Science, College of Agriculture, Fujian Agriculture and Forestry University, Fuzhou, China; ^2^ Fujian Provincial Key Laboratory of Haixia Applied Plant Systems Biology, College of Life Science, College of Agriculture, Fujian Agriculture and Forestry University, Fuzhou, China; ^3^ College of Life Science, Industry and University Research Cooperation Demonstration Base in Fujian Province, Ningde Normal University, Ningde, China

**Keywords:** kenaf, COMT, lignin synthesis, salt stress, expression pattern

## Abstract

Caffeic acid O-methyltransferase (*COMT*) catalyzes the penultimate methylation in monolignol biosynthesis, controlling lignin composition and abiotic-stress tolerance. Kenaf (*Hibiscus cannabinus* L.), a fast bast-fiber crop rich in lignin, is valued for its mechanical strength and resilience to salinity. However, the *COMT* gene family has not yet been systematically characterized in this species. Here, we integrated phylogenetics, synteny, promoter and transcriptome analyses to create a comprehensive profile of kenaf COMT genes. Genome-wide screening identified 81 *HcCOMT* genes. Phylogenetic reconstruction with COMTs from *Arabidopsis thaliana* and *Gossypium hirsutum* resolved 10 distinct clades. Synteny analysis revealed 2 collinear blocks with Arabidopsis and 14 with cotton, whereas intraspecific duplication events indicated recent lineage-specific expansion. Promoter analysis identified numerous cis-elements responsive to light, phytohormones and abiotic stress, suggesting complex transcriptional regulation. Transcriptome mining uncovered 6 candidate genes with pronounced tissue specificity and salt responsiveness; qRT-PCR confirmed these patterns in root, stem and leaf tissues under 200 mM NaCl: *HcCOMT28* and *HcCOMT29* were repressed in the leaf, whereas *HcCOMT11*, *HcCOMT12*, *HcCOMT13*, and *HcCOMT17* were up-regulated, consistent with altered lignin deposition patterns. Our findings provide a comprehensive genomic resource delineating the structure, evolution, and salt-responsive expression of the kenaf *COMT* family, and establish a foundation for elucidating the molecular mechanisms underlying lignin-mediated salt tolerance and for breeding elite kenaf cultivars with tailored fiber properties.

## 1 Introduction

Lignin is the principal hydrophobic polymer that impacts mechanical rigidity and hydrophobicity to secondary-thickened plant cell walls. It is synthesized from three hydroxylated and methoxylated phenylpropane alcohols: p-coumaryl (H), coniferyl (G) and sinapyl (S) alcohols, via oxidative coupling reactions that are catalyzed predominantly by class III peroxidases and laccases (Tu et al., 2010; Liu et al., 2018). By encrusting the polysaccharide matrix, lignin reinforces vascular tissues, enables long-distance water transport, and erects a physical barrier against insects, pathogens and abiotic stresses, thereby enhancing lodging resistance and stress tolerance (Moura et al., 2010; Liu et al., 2018). Biosynthetically, lignin originates from phenylalanine through the general phenylpropanoid pathway, followed by a series of hydroxylation, O-methylations, CoA-ligations, and NADPH-dependent reductions that generate the three monolignols (Vanholme et al., 2019). These monomers are exported to the apoplast, where they undergo radical coupling to form the heterogeneous lignin polymer embedded in the secondary cell wall (Vanholme et al., 2010; Abdelaziz et al., 2016; Tuskan et al., 2019; Vanholme et al., 2019). Among the genes governing lignin composition, caffeoyl-CoA 3-O-methyltransferase (COMT) is a pivotal O-methyltransferase that channels metabolic flux towards S-lignin by catalyzing the methylation of 5-hydroxyconiferyl alcohol and its aldehyde/CoA esters (Vanholme et al., 2019; Wu et al., 2021). Alteration in COMT activity can shift lignin composition and architecture, affecting hydrophobicity, elasticity, and cation exchange potential of the cell wall. In the context of salt stress, enhanced S-unit lignin deposition has been associated with reduced Na+ influx, improved water-use efficiency, and stabilization of cell wall structure under osmotic pressure (Moura et al., 2010; Vanholme et al., 2019). Recent studies further demonstrate that COMT expression is dynamically regulated under both salinity and drought, directly linking lignin remodeling to stress resilience mechanisms (Huang et al., 2024). Collectively, these findings underscore COMT as a mechanistic bridge between lignin biochemistry and salt tolerance, providing a molecular target for engineering stress-resilient crops. All COMTs share a conserved C-terminal methyltransf-2 domain harboring the S-adenosyl-L-methionine (SAM)/SAH-binding pocket and the substrate-binding site, where the N-terminal helical domain, composed of nine heptad repeats, mediates homo- and heterodimerization that is essential for efficient catalysis and protein-protein interactions (Vanholme et al., 2010; Zagel et al., 2013).

COMT genes have undergone dynamic evolution driven by whole-genome duplication (WGD) and dispersed duplication events (Huang et al., 2013; Liu et al., 2021). Since the first report of a *COMT* multigene family in *Populus tremuloides* (Bugos et al., 1991), extensive genome-wide surveys have revealed substantial family expansions: 33 *COMT* gene members identified in *Oryza sativa* (Liang et al., 2022), 7 members in *Eucalyptus grandis* (Carocha et al., 2015), 23 members in *Catalpa bungei* (Lu et al., 2019), 92 in blueberries (Liu et al., 2021), 25 in *Populus trichocarpa* (Shi et al., 2010), and 25 in *Betula pendula* (Chen et al., 2020b). Functionally, *COMT* show maximal activity in lignifying tissues, such as stem segments display the highest transcript levels (Lv et al., 2011). RNA-mediated suppression of *COMT* in *Brassica napus* decreases stem lignin without compromising seed oil quality (Oraby and Ramadan, 2014), whereas its overexpression in Arabidopsis promotes biomass accumulation and salt tolerance through enhanced melatonin biosynthesis (Lee et al., 2014; Yang et al., 2019). Moreover, *Sorghum bicolor COMT* participates in tricin (a flavone lignin monomer) biosynthesis by methylating luteolin and selgin (Eudes et al., 2017), and Populus *COMT4* alters lignin crosslinking by increasing G-unit incorporation (Cai et al., 2016). Despite these advances, the *COMT* gene family remains uncharacterized in kenaf (*Hibiscus cannabinus* L.).

Kenaf (*Hibiscus cannabinus* L.), belonging to the Malvaceae family, is an industrial crop holding high cellulosic fiber and lignin content, predominantly grown in Asia and Africa (Ayadi et al., 2011; Niu et al., 2017). Kenaf is an appealing fiber and lignin source for paper manufacture, owing to its environmentally friendly nature, such as biodegradability, renewability, and low energy consumption (Bhardwaj et al., 2005; Villar et al., 2009; Niu et al., 2017). It also has great potential for utilization in oil absorption, animal feed, and value-added industrial products (Ayadi et al., 2011; Niu et al., 2017). Moreover, it is reported that kenaf can survive in water-deficient areas and adapt well to diverse adverse stresses, such as high salinity, drought and extreme temperature (Bhardwaj et al., 2005; Niu et al., 2017). Its inherent tolerance to drought, salinity, and extreme temperatures makes it an ideal model for dissecting fiber development and stress adaptation. However, the evolutionary relationship, structural features, and functional divergence of the COMT family in *H. cannabinus* have not yet been investigated.

Here, we performed a comprehensive genome-wide identification and characterization of the *COMT* gene family in kenaf. We reconstructed the phylogeny of COMT proteins across land plants, investigated the duplication history and conserved motifs of HcCOMT members, and profiled their transcriptional responses to salt stress in the salt-tolerant cultivar Fuhong18 and the salt-sensitive cultivar Zanyin1, and detected potential interactions among HcCOMT members using protein-protein interaction assays. These findings establish a foundation for elucidating the molecular mechanisms underlying lignin deposition and stress adaptation in kenaf and provide valuable genetic resources for fiber crop improvement.

## 2 Materials and methods

### 2.1 Identification and characteristics of *COMT* genes in kenaf

The kenaf (*Hibiscus cannabinus* L.) genome assembly (Accession: GWHACDB00000000.1, BioProject: PRJCA000871, BioSample: SAMC036340, released on 1 July 2021) together with its corresponding CDS and protein sequences were retrieved from Genome Warehouse of the BIG Data Center (BIGD, https://bigd.big.ac.cn/gwh). To obtain a comprehensive COMT gene set, we integrated homology- and domain-based searches. First, the 16 functionally characterized COMT proteins from *Arabidopsis thaliana* were used as queries to identify homologous genes using a BLASTp search (BLOSUM62 matrix, E-value ≤ 1 ×10^-5^) against the kenaf proteome. Putative hits were subsequently screened for the presence of the methyltransf-2 domain (PF00891) using the Hidden Markov Model (HMM) profile downloaded from the Pfam database (http://pfam.xfam.org). All candidates were further validated with the NCBI-CDD (https://www.ncbi.nlm.nih.gov/cdd) and SMART (http://smart.embl-heidelberg.de/) to confirm domain integrity. Proteins lacking the canonical SAM-binding motif and substrate-binding pocket were discarded. Physicochemical parameters: such as molecular weight (MW), isoelectric point (pI), and grand average of hydrophilicity (GRAVY), which are valuable for hypothesizing about its functional roles, potential interaction partners, and its suitability for specific cellular environments, were calculated with the ExPASy ProtParam server (https://www.expasy.org/resources/compute-pI-mw). Subcellular localization of HcCOMT were predicted independently by WOLF-PSORT (http://wolfpsort.hgc.jp/) and CELLO (http://cello.life.nctu.edu.tw/) under the “plant” settings; only consensus predictions were retained.

### 2.2 Phylogenetics, gene structure, and chromosomal distribution

To assess the evolutionary conservation and functional divergence of COMT across taxa, full-length COMT proteins from three representative plant species were aligned with MAFFT software (Katoh and Standley, 2013). A Maximum Likelihood (ML) phylogeny was then inferred with IQ-TREE (v3.0.0) using 1000 bootstrap replicates to estimate node support. Exon-intron architectures of *HcCOMTs* were visualized with GSDS 2.0 (http://gsds.gao-lab.org) using the corresponding genome annotation file. Conserved motifs (width 6–50 aa, maximum 15 motifs) were identified by MEME (http://meme-suite.org/meme/) and mapped onto the phylogeny. Secondary structures of HcCOMTs were predicted by PSIPRED workbench (https://bioinf.cs.ucl.ac.uk/psipred/), tertiary topology homology models were generated by SWISS-MODEL, and model quality was assessed by QMEAN and PROCHECK (Biasini et al., 2014). Chromosomal locations were extracted from the genome GFF and plotted with MapChart 2.2 software (Voorrips, 2002). For promoter analysis, 2000 bp upstream of the translation start codon of each *HcCOMT* was scanned against the PlantCARE database to identify cis-regulatory elements responsive to phytohormones and abiotic stresses (Lescot et al., 2002). GO enrichment analysis was used agriGO v 2.0 database (http://systemsbiology.cau.edu.cn/agriGOv2/index.php) to identify significant GO terms among the COMT-associated genes. Statistical significance was assessed using the hypergeometric test with a Benjamini-Hochberg false discovery rate (FDR) correction, and terms with FDR-adjusted p < 0.05 were considered significantly enriched. The background set consisted of all annotated genes in the kenaf genome.

### 2.3 Gene duplication and synteny analysis

Paralogous pairs were identified via an all-vs-all BlastP (E-value ≤ 1 ×10^-10^) followed by MCScanX (http://chibba.pgml.uga.edu/mcscan2/) with default parameters (Wang et al., 2012, 2024). Segmental and tandem duplications were classified according to the criteria of Guo et al. (2013). Synteny blocks between *H. cannabinus*, *Arabidopsis thaliana*, and *Gossypium hirsutum* were inferred by TBtools software (v 1.092) using protein identity ≥ 70% and coverage ≥ 80% (Chen et al., 2020a). Non-synonymous substitution rate (Ka), synonymous substitution rate (Ks) value, and Ka/Ks ratios for the paralogs *COMT* gene pairs were calculated by the KaKs-Calculator implemented in TBtools. Divergence time (T) was estimated under a neutral evolutionary rate of 1.5 × 10–^8^ substitutions site^-1^ year^-1^ for dicots (Koch et al., 2003).

### 2.4 Protein-protein interaction prediction

Putative functional partnerships of HcCOMT proteins were inferred with the STRING database (https://string-db.org/), with *Gossypium hirsutum* selected as the reference species due to its close phylogenetic relationship to kenaf. Only interactions with a confidence score threshold of 0.9 were retained to minimize false positives.

### 2.5 Plant materials and salt-stress treatments

Two kenaf varieties, salt-tolerant cultivar Fuhong18 and salt-sensitive cultivar Zanyin1, were used for salt stress treatments. After surface-sterilization, seeds were germinated on moist filter paper and transferred to nutritional soil (pH 6.5 – 7.0, electrical conductivity of approximately 1.2 – 1.5 dS/m, and an organic matter content of around 2.0–2.5%). Seedlings were grown in an artificial climate chamber (28 °C, 16 h photoperiod with light intensity 300 µmol·m^−2^ s^−1^, relative humidity 50 ± 10%). At the 5-leaf-stage, uniform seedlings were irrigated with 200 mM NaCl solution until the soil reached field capacity. Plants were then maintained under the same salinity regime by watering with the same NaCl solution at regular intervals to sustain a stable salt-stress environment, and control plants received water only. Leaves, stems, and roots were harvested at 0-, 7-, 14-, and 21-days post-treatment. Three biological replicates per tissue per time-point were snap-frozen in liquid nitrogen and stored at − 80 °C.

### 2.6 RNA extraction and quantitative real-time PCR

Total RNA was isolated using the RNAprep Pure Plant Kit (Tiangen, Beijing, China) following the manufacture’s protocol for polysaccharide-rich tissues. RNA integrity was verified by 1.2% agarose gel electrophoresis and quantified on a Nanodrop 2000C (Thermo). First-strand cDNA was synthesized from 2 µg DNase-treated RNA with the PrimeScript RT Reagent kit (Takara, Japan). Gene-specific primers (**Supplementary Table S1**) were designed with Primer-BLAST (NCBI) to span exon-exon junctions, and primer specificity was further confirmed by BLAST analysis and PCR amplification to ensure unique target amplification; the kenaf PP2A gene served as the endogenous control (Niu et al., 2017). qRT-PCR was performed in triplicate on an ABI 7500 Fast System (Applied Biosystems, Waltham, MA, USA) using SYBR Premix Ex Taq (Takara) in 20 µL reactions including 2 × SYBR Premix Ex Taq 10 µL, 10 μM forward primer 0.5 µL, 10 μM reverse primer 0.5 µL, cDNA template 1 µL and ddH_2_O 8 µL. PCR amplification was set at 95 °C for 30 s, followed by 40 cycles of 95 °C for 5 s and 60 °C for 20 s. Melting-curve analysis confirmed amplicon specificity. Relative expression was calculated by the 2^−ΔΔCt^ method and normalized to PP2A. Statistical significance was assessed by one-way ANOVA followed by Tukey’s HSD test (*p* < 0.05).

## 3 Results

### 3.1 Identification and physicochemical properties of kenaf *COMT* genes

A stringent pipeline combining HMM profile and BLAST-based searches was used to mine the kenaf genome for *COMT* genes in kenaf. An initial HMM scan with the methyltransf-2 domain (PF00891) yielded 93 putative HcCOMT proteins, after removal of truncated and artefactual sequences by CDD and SMART validation, 81 high-confidence loci were retained. These *HcCOMT* genes were designated *HcCOMT01* - *HcCOMT81* according to their positions on the chromosome (**Table 1**, **Supplementary Figure S1**). The deduced proteins range from 100 (HcCOMT78) to 1069 amino acids (HcCOMT13), with corresponding molecular weights of 11.21 kDa - 122.39 kDa and theoretical isoelectric points (pI) spanning from 4.65 to 9.58. Fourteen proteins are predicted to be unstable (Instability Index > 40), whereas 71.6% are hydrophilic with GRAVY < 0. Subcellular prediction with CELLO and WoLF PSORT suggests predominant cytoplasmic localization (72.8%), followed by cytoskeletal association (11.1%). Six proteins (HcCOMT13, HcCOMT30, HcCOMT54, HcCOMT61, HcCOMT64, and HcCOMT80) are predicted to enter the nucleus; five (HcCOMT10, HcCOMT36, HcCOMT60, HcCOMT70, and HcCOMT76) are plastid; and single representatives are targeted to mitochondria (HcCOMT2) or the endoplasmic reticulum (HcCOMT23) (**Table 1**).

**Table 1:** The *COMT* gene family members and their physicochemical property charateristics in kenaf.

Gene Name	Gene ID	Chromosome	Amino acid Length	Molecular Weight/kDa	Theoretical pI	Instability Index	Grand Average of Hydropathicity	Subcellular Localization
*HcCOMT01*	Hc.01G038880	1	358	39.23	5.83	25.03	-0.089	Cytoplasmic
*HcCOMT02*	Hc.01G038910	1	236	26.34	8.51	36.14	-0.236	Mitochondrial
*HcCOMT03*	Hc.01G041930	1	740	83.23	5.17	34.23	-0.089	Cytoplasmic
*HcCOMT04*	Hc.01G052160	1	352	38.65	5.46	45.68	-0.030	Cytoskeleton
*HcCOMT05*	Hc.02G001610	2	349	38.29	5.58	33.14	0.033	Cytoplasmic
*HcCOMT06*	Hc.02G036860	2	351	39.11	5.18	41.09	-0.061	Cytoskeleton
*HcCOMT07*	Hc.03G006410	3	357	39.24	5.80	39.77	-0.043	Cytoskeleton
*HcCOMT08*	Hc.03G006420	3	221	24.27	6.05	28.93	0.038	Cytoplasmic
*HcCOMT09*	Hc.04G005500	4	355	38.93	5.51	38.02	-0.027	Cytoskeleton
*HcCOMT10*	Hc.04G005880	4	265	29.46	7.42	29.74	0.108	Chloroplast
*HcCOMT11*	Hc.04G034230	4	366	40.50	5.54	29.68	-0.007	Cytoplasmic
*HcCOMT12*	Hc.06G001820	6	507	56.53	4.65	34.33	-0.246	Cytoskeleton
*HcCOMT13*	Hc.06G002190	6	1069	122.39	7.06	43.59	-0.260	Nuclear
*HcCOMT14*	Hc.06G002200	6	323	35.77	5.26	49.35	0.007	Cytoplasmic
*HcCOMT15*	Hc.07G036520	7	412	45.92	5.32	28.13	-0.069	Cytoplasmic
*HcCOMT16*	Hc.07G036530	7	367	40.80	5.45	30.84	-0.046	Cytoskeleton
*HcCOMT17*	Hc.08G005760	8	367	40.60	5.50	35.41	-0.034	Cytoplasmic
*HcCOMT18*	Hc.09G027970	9	362	40.70	5.30	40.16	-0.024	Cytoplasmic
*HcCOMT19*	Hc.09G028000	9	755	83.75	5.46	32.35	0.021	Cytoplasmic
*HcCOMT20*	Hc.09G028030	9	364	40.62	5.91	24.89	-0.052	Cytoplasmic
*HcCOMT21*	Hc.09G028050	9	362	40.49	5.35	32.24	-0.052	Cytoskeleton
*HcCOMT22*	Hc.09G028060	9	540	60.54	5.34	27.37	-0.011	Cytoplasmic
*HcCOMT23*	Hc.09G028070	9	248	27.19	5.52	29.79	0.342	Endoplasmic reticulum
*HcCOMT24*	Hc.09G028090	9	365	40.79	5.21	31.69	-0.023	Cytoplasmic
*HcCOMT25*	Hc.09G028110	9	355	39.53	5.15	30.55	-0.071	Cytoplasmic
*HcCOMT26*	Hc.09G028130	9	386	43.07	6.03	31.11	-0.024	Cytoplasmic
*HcCOMT27*	Hc.09G028150	9	359	40.16	5.10	30.83	-0.105	Cytoplasmic
*HcCOMT28*	Hc.10G012630	10	357	39.62	5.88	31.82	0.051	Cytoskeleton
*HcCOMT29*	Hc.10G014360	10	426	47.54	5.98	40.61	-0.031	Cytoplasmic
*HcCOMT30*	Hc.10G025220	10	285	31.45	5.60	42.04	-0.115	Nuclear
*HcCOMT31*	Hc.11G032250	11	365	39.95	5.42	26.85	0.003	Cytoplasmic
*HcCOMT32*	Hc.13G000410	13	346	38.34	6.23	31.31	-0.108	Cytoplasmic
*HcCOMT33*	Hc.13G010150	13	357	40.44	5.64	44.44	-0.138	Cytoplasmic
*HcCOMT34*	Hc.14G013890	14	364	40.37	6.05	30.94	0.041	Cytoplasmic
*HcCOMT35*	Hc.14G016650	14	391	43.87	6.23	35.43	-0.039	Cytoplasmic
*HcCOMT36*	Hc.14G019970	14	298	32.41	6.45	43.15	-0.041	Chloroplast
*HcCOMT37*	Hc.14G021480	14	428	47.78	6.94	27.43	-0.007	Cytoplasmic
*HcCOMT38*	Hc.14G021490	14	363	40.93	5.54	32.22	-0.016	Cytoplasmic
*HcCOMT39*	Hc.14G021510	14	364	40.75	6.06	33.47	-0.010	Cytoplasmic
*HcCOMT40*	Hc.14G021520	14	365	40.67	5.22	31.54	0.002	Cytoplasmic
*HcCOMT41*	Hc.14G021670	14	442	49.13	5.61	28.23	0.052	Cytoplasmic
*HcCOMT42*	Hc.14G021690	14	365	40.22	5.09	32.71	0.095	Cytoplasmic
*HcCOMT43*	Hc.14G021710	14	576	64.88	8.09	34.90	-0.087	Cytoplasmic
*HcCOMT44*	Hc.14G021740	14	365	40.50	5.18	29.50	0.030	Cytoplasmic
*HcCOMT45*	Hc.14G021760	14	415	46.41	5.33	33.02	0.017	Cytoskeleton
*HcCOMT46*	Hc.14G021770	14	222	24.64	5.51	26.90	-0.019	Cytoplasmic
*HcCOMT47*	Hc.14G021790	14	356	39.52	5.28	26.31	0.096	Cytoplasmic
*HcCOMT48*	Hc.14G021800	14	359	39.61	4.94	28.24	0.048	Cytoplasmic
*HcCOMT49*	Hc.14G021810	14	363	40.91	5.38	29.35	-0.017	Cytoplasmic
*HcCOMT50*	Hc.14G021820	14	365	40.82	5.65	33.77	-0.018	Cytoplasmic
*HcCOMT51*	Hc.14G021840	14	365	40.80	5.65	33.77	-0.011	Cytoplasmic
*HcCOMT52*	Hc.14G021850	14	365	40.80	5.65	33.77	-0.011	Cytoplasmic
*HcCOMT53*	Hc.14G021870	14	365	40.80	5.65	33.77	-0.011	Cytoplasmic
*HcCOMT54*	Hc.14G021890	14	969	106.61	5.40	46.84	-0.250	Nuclear
*HcCOMT55*	Hc.14G021930	14	365	40.82	5.75	32.33	-0.012	Cytoplasmic
*HcCOMT56*	Hc.14G021940	14	365	40.82	5.75	33.92	-0.034	Cytoplasmic
*HcCOMT57*	Hc.14G021960	14	698	79.03	5.45	42.46	-0.235	Cytoplasmic
*HcCOMT58*	Hc.14G021990	14	372	41.57	5.85	31.25	-0.105	Cytoplasmic
*HcCOMT59*	Hc.14G022010	14	495	54.90	6.32	24.46	-0.116	Cytoplasmic
*HcCOMT60*	Hc.14G026550	14	772	86.47	5.80	41.69	-0.095	Chloroplast
*HcCOMT61*	Hc.15G011420	15	331	36.59	9.58	51.75	-0.198	Nuclear
*HcCOMT62*	Hc.15G011710	15	375	41.12	5.95	32.33	0.047	Cytoplasmic
*HcCOMT63*	Hc.15G011720	15	365	39.81	5.44	25.47	0.030	Cytoplasmic
*HcCOMT64*	Hc.15G011730	15	860	94.03	5.70	26.82	-0.025	Nuclear
*HcCOMT65*	Hc.15G011850	15	365	39.92	5.46	25.55	-0.021	Cytoplasmic
*HcCOMT66*	Hc.15G011860	15	365	39.79	5.44	23.95	0.019	Cytoplasmic
*HcCOMT67*	Hc.15G016160	15	266	29.88	5.26	36.41	0.073	Cytoplasmic
*HcCOMT68*	Hc.15G020190	15	375	41.61	5.71	39.25	-0.035	Cytoplasmic
*HcCOMT69*	Hc.15G024170	15	371	40.66	5.45	39.63	-0.097	Cytoplasmic
*HcCOMT70*	Hc.15G024180	15	313	34.92	8.78	30.53	-0.080	Chloroplast
*HcCOMT71*	Hc.16G006110	16	390	43.15	5.83	28.55	-0.009	Cytoplasmic
*HcCOMT72*	Hc.16G006600	16	459	50.79	5.71	31.62	-0.098	Cytoplasmic
*HcCOMT73*	Hc.16G006660	16	283	31.48	5.38	27.13	-0.165	Cytoplasmic
*HcCOMT74*	Hc.16G006680	16	342	37.88	5.48	27.62	-0.070	Cytoplasmic
*HcCOMT75*	Hc.16G006700	16	356	39.14	5.66	28.97	0.039	Cytoplasmic
*HcCOMT76*	Hc.16G006720	16	353	38.70	5.09	31.99	0.020	Chloroplast
*HcCOMT77*	Hc.16G009710	16	358	39.69	5.55	39.32	-0.193	Cytoplasmic
*HcCOMT78*	Hc.16G027350	16	100	11.21	6.57	36.20	-0.231	Cytoplasmic
*HcCOMT79*	Hc.17G002150	17	185	20.29	4.91	38.47	0.154	Cytoplasmic
*HcCOMT80*	Hc.17G022820	17	371	41.39	5.30	43.24	-0.179	Nuclear
*HcCOMT81*	Hc.17G023110	17	365	39.76	5.25	28.17	0.005	Cytoplasmic

### 3.2 Phylogeny and molecular evolution of *HcCOMT* genes

**Figure 1:**
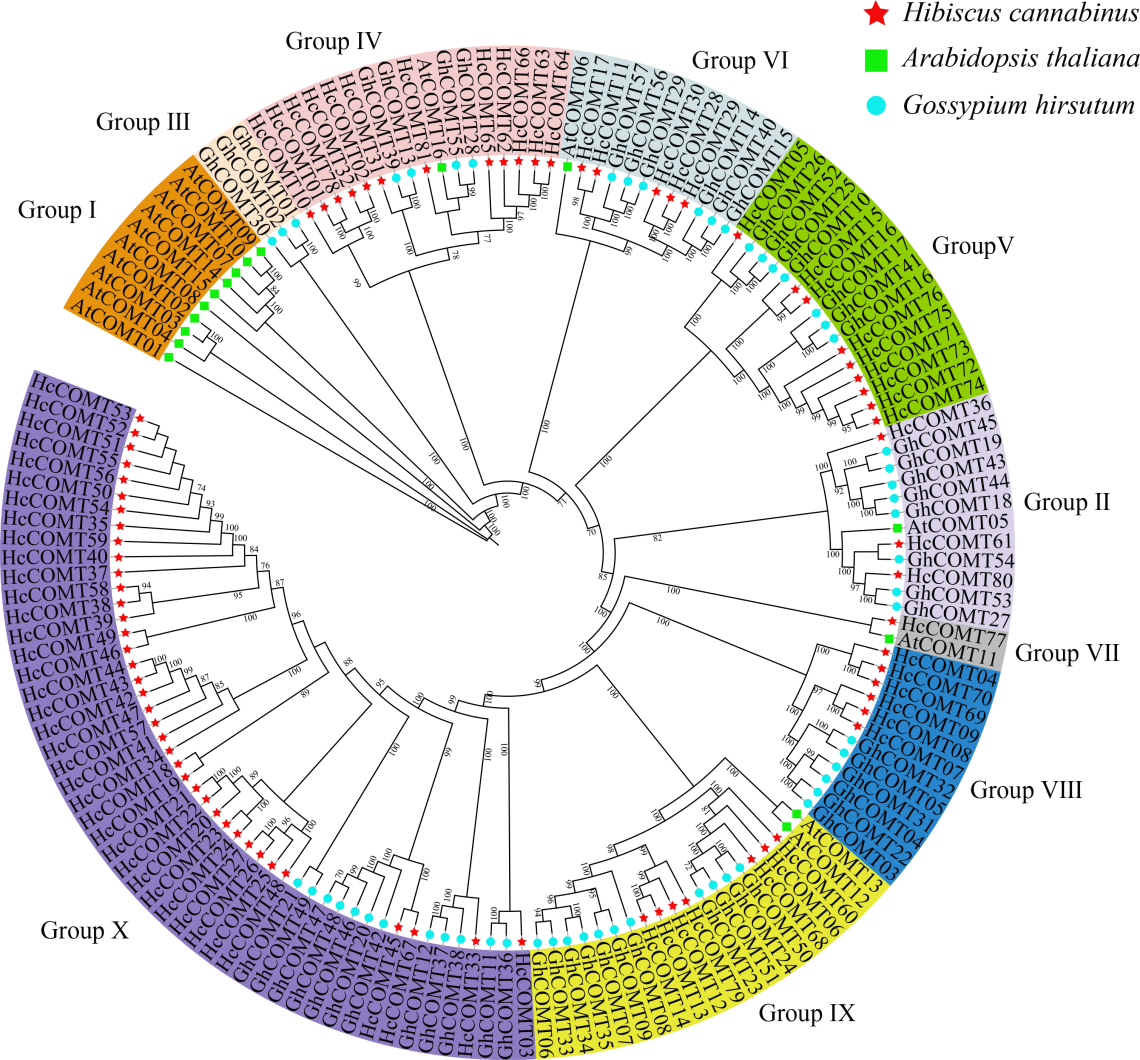
Phylogenetic tree for *COMT* gene family in three species. This unrooted tree was built using the COMT sequences of *Hibiscus cannabinus* (red stars), *Arabidopsis thaliana* (green ssquares) and *Gossypium hirsutum* (blue dots) by Maximum Likelihood (ML) method. *COMT* genes were divided into 10 groups (Group I-X) according to the clades and bootstrap values.

To resolve the evolutionary relationships among *COMT* genes, we assembled a data set of 123 full-length COMT proteins from *Hibiscus cannabinus* (81), *Gossypium hirsutum* (26) and *Arabidopsis thaliana* (16). A Maximum Likelihood (ML) phylogeny robustly partitioned the family into ten clades (I-X) supported by ≥ 70% bootstrap values (**Figure 1**, **Supplementary Table S2**). Clades V and X are the largest groups with more than 20 COMT members, whereas clades I, III, and VII contained less than 10 members. Notably, kenaf contributes 34 members to the species-specific *COMT* clade X, whereas Arabidopsis members are restricted to clade I, and cotton members dominate clade III. These topologies suggest lineage-specific expansion and functional divergence following the divergence of the Malvaceae and Brassicaceae.

### 3.3 Motif composition and gene structure of the kenaf *COMT* genes

**Figure 2:**
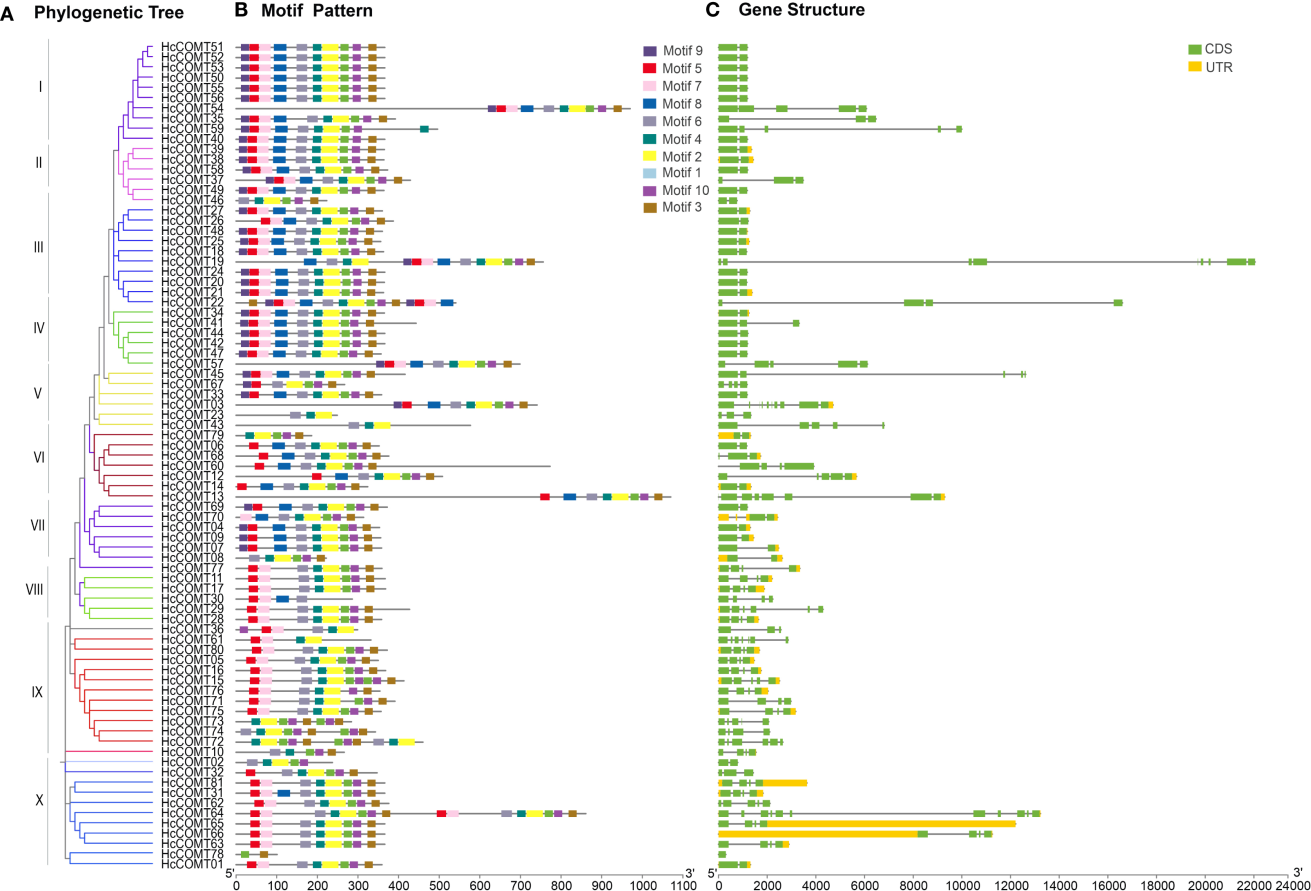
Gene structure characteristics analysis of *HcCOMT* genes. **(A)** The NJ tree on the left comprised of 81 *COMT* genes from kenaf. **(B)** The conserved motifs were indicated by the different colors boxes. **(C)** Gene structures of *HcCOMT* genes, the green color indicates the CDS regions, the yellow color indicates the 5’ and 3’-UTR regions, and the gray lines indicate the introns.

To gain a better understanding of the structural characteristics of HcCOMT proteins, the compositions of conserved motifs were analyzed using MEME. Ten conserved motifs, labeled as motifs 1–10, were identified. With the exception of a few outliers, all proteins in clades I-IV harbor the complete motif complement (motifs 1–10). Progressive motif loss is evident in more derived clades: *HcCOMT46* (clade II) lacks motif 5, 7, 8, and 9; *HcCOMT26* (clade III) lacks motif 9; *HcCOMT67* (clade V) missed motif 7, 8, and 4; *HcCOMT23* and *HcCOMT43* (clade V) missed the motif 5, 7, 8, 9, 1, 10 and 3; and *HcCOMT79* (clade VI) missed the motif 5, 6, 7, 8, and 9. The other members (*HcCOMT06*, *HcCOMT68*, *HcCOMT60*, *HcCOMT12*, *HcCOMT14*, and *HcCOMT13*) in the clade VI lacks the motif 9 and 7. While members in the clade VII miss motifs 7; clade VIII branch 9 and 8; and Clade X member *HcCOMT78* retains only motifs 1 and 3. The presence of duplicated motif blocks in *HcCOMT64* suggests a recent internal duplication event (**Figures 2A, B**). The type of conserved motifs in each subfamily were basically the same, indicating that the same subfamily was composed of similar conserved structural domains and may have similar biological functions. Intron-exon organization pattern mirrors the phylogenetic architecture. Most members of clades I-IV (such as *HcCOMT51*, *HcCOMT40*, *HcCOMT27*, and *HcCOMT34*) possess a single intron, whereas clades V-X (such as *HcCOMT33*, *HcCOMT79*, *HcCOMT69*, *HcCOMT10*, and *HcCOMT01*) display three to five introns, and *HcCOMT64* contained 10 introns and *HcCOMT78* has no intron. Exceptionally, *HcCOMT19* contains 8 exons separated by two unusually long introns, a feature consistent with its isolated phylogenetic position (**Figure 2C**).

### 3.4 Chromosomal distribution and collinearity analysis of *HcCOMT* genes

To illustrate the evolutionary events of *HcCOMT* genes, the gene location on the chromosome of kenaf was investigated based on their genome sequences. The result revealed that 81 *HcCOMT* members were randomly distributed across eighteen chromosomes. Among them, Chr2, Chr3, Chr7, and Chr13 contained two *HcCOMT* genes; Chr4, Chr6, Chr10, and Chr17 had three genes; Chr8 and Chr11 each had one gene. Chromosome 14 is the major reservoir (27 *HcCOMT* genes), whereas chromosomes 5, 12, and 18 lack COMT loci entirely (**Table 1**, **Supplementary Figure S1**).

**Figure 3:**
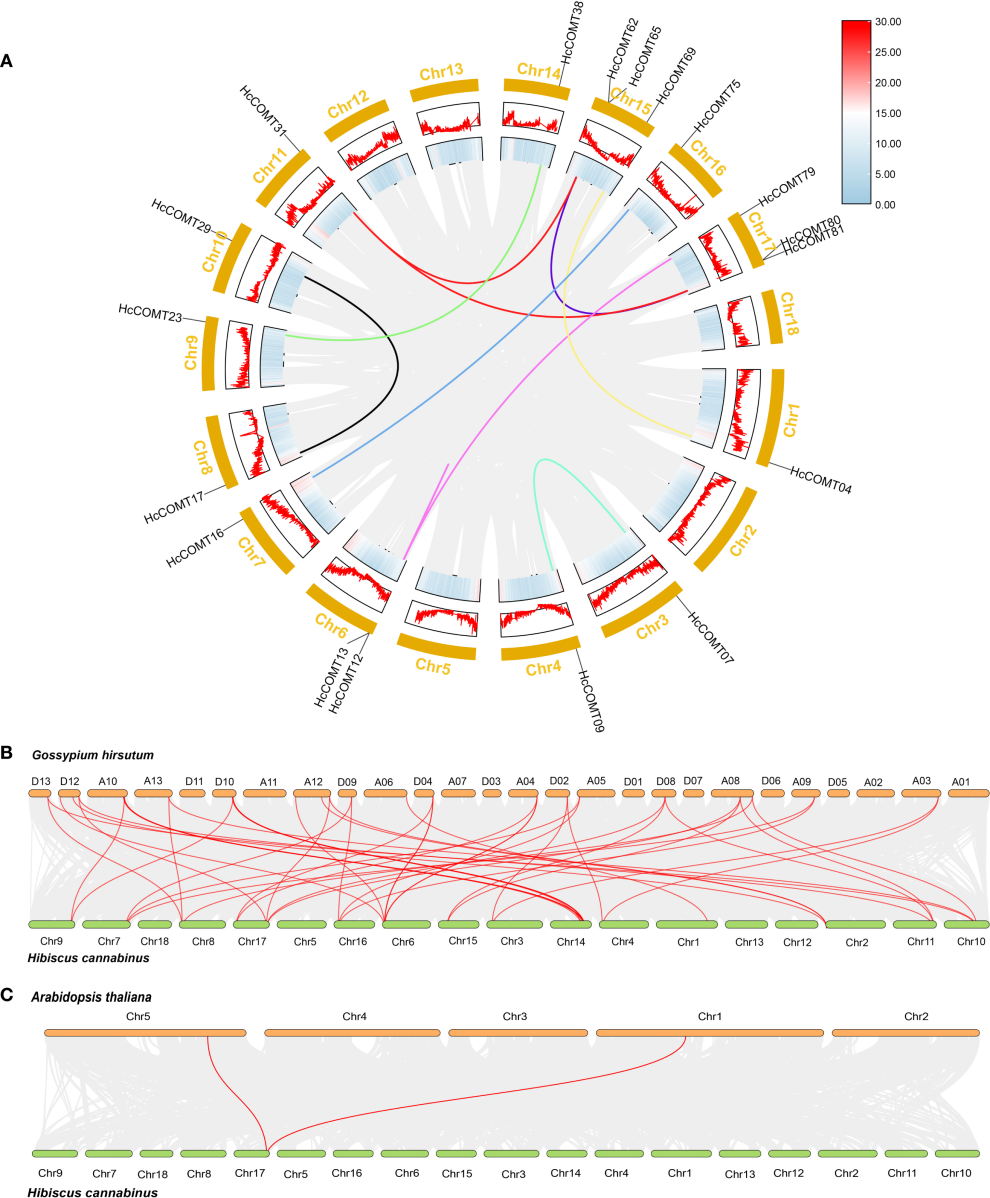
Synteny analysis of *HcCOMT* genes in intra- and inter- species. **(A)** Gene duplication of *COMT* genes in kenaf. The gray background lines represent all the syntenic blocks in kenaf genome, and the color lines represent the segmental or tandem duplication blocks among *HcCOMT* genes. **(B–C)** Multiple collinearity analysis of *COMT* genes among *Arabidopsis*, kenaf, and cotton genome. The red lines show the kenaf *COMT* genes orthologous in *Arabidopsis* and cotton, the gray lines show the collinear blocks background among these species genomes.

To elucidate the evolutionary relationships among *HcCOMT* genes, we analyzed the synchrony within the *HcCOMT* gene family. The results showed that segment replication accounts for 14 paralogous pair (*HcCOMT31*/*HcCOMT62*, *HcCOMT31*/*HcCOMT65*, *HcCOMT31*/*HcCOMT81*, *HcCOMT31*/*HcCOMT80*, *HcCOMT29*/*HcCOMT17*, *HcCOMT23*/*HcCOMT38*, *HcCOMT16/HcCOMT75*, *HcCOMT12*/*HcCOMT79*, *HcCOMT07*/*HcCOMT09*, *HcCOMT04*/*HcCOMT69*, *HcCOMT80*/*HcCOMT62*, *HcCOMT80*/*HcCOMT65*, *HcCOMT81*/*HcCOMT62*, and *HcCOMT81*/*HcCOMT65*), and three tandem arrays (*HcCOMT62*/*HcCOMT65*, *HcCOMT80*/*HcCOMT81*, and *HcCOMT12*/*HcCOMT13*) were identified (**Figure 3A**). All paralogous pairs exhibit Ka/Ks ratios less than 1, indicating strong purifying selection during the evolution of *HcCOMT* genes (**Supplementary Table S3**).

To gain further insight into the evolutionary relationships of *HcCOMTs*, we constructed interspecies comparative syntenic maps involving kenaf, Arabidopsis, and cotton. The results revealed that 2 orthologous pairs exhibited syntenic relationships between kenaf and Arabidopsis (*HcCOMT81*/*AtCOMT15* and *HcCOMT80*/*AtCOMT5*) (**Figure 3C**, **Supplementary Table S3**). While 49 orthologous pairs (*HcCOMT17*/*GhCOMT56*, *HcCOMT29*/*GhCOMT56*, *HcCOMT05*/*GhCOMT52*, *HcCOMT12*/*GhCOMT50*, *HcCOMT31*/*GhCOMT53*, *HcCOMT80*/*GhCOMT53*, *HcCOMT18*/*GhCOMT21*, *HcCOMT55*/*GhCOMT20*, *HcCOMT58*/*GhCOMT21*, *HcCOMT37*/*GhCOMT20*, *HcCOMT40*/*GhCOMT21*, *HcCOMT36*/*GhCOMT18*, *HcCOMT17*/*GhCOMT29*, *HcCOMT29*/*GhCOMT29*, *HcCOMT24*/*GhCOMT47*, *HcCOMT55*/*GhCOMT46*, *HcCOMT56*/*GhCOMT47*, *HcCOMT37*/*GhCOMT46*, *HcCOMT40*/*GhCOMT49*, *HcCOMT36*/*GhCOMT43*, *HcCOMT05*/*GhCOMT25*, *HcCOMT05*/*GhCOMT27*, *HcCOMT12*/*GhCOMT23*, *HcCOMT80*/*GhCOMT27*, *HcCOMT15*/*GhCOMT41*, *HcCOMT75*/*GhCOMT41*, *HcCOMT03*/*GhCOMT11*, *HcCOMT13*/*GhCOMT33*, *HcCOMT12*/*GhCOMT33*, *HcCOMT79*/*GhCOMT33*, *HcCOMT13*/*GhCOMT06*, *HcCOMT12*/*GhCOMT06*, *HcCOMT79*/*GhCOMT06*, *HcCOMT07*/*GhCOMT31*, *HcCOMT09*/*GhCOMT31*, *HcCOMT15*/*GhCOMT10*, *HcCOMT75*/*GhCOMT10*, *HcCOMT31*/*GhCOMT39*, *HcCOMT62*/*GhCOMT39*, *HcCOMT81*/*GhCOMT39*, *HcCOMT17*/*GhCOMT14*, *HcCOMT29*/*GhCOMT14*, *HcCOMT31*/*GhCOMT13*, *HcCOMT62*/*GhCOMT13*, *HcCOMT81*/*GhCOMT13*, *HcCOMT15*/*GhCOMT16*, *HcCOMT75*/*GhCOMT16*, *HcCOMT07*/*GhCOMT03*, and *HcCOMT09*/*GhCOMT03*) between kenaf and cotton (**Figure 3B**, **Supplementary Table S4**). The extensive micro-synteny with G. hirsutum confirms the close evolutionary relationship within the Malvaceae and underscores the role of shared WGD events in shaping the COMT repertoire.

### 3.5 Cis-regulatory elements prediction of the kenaf *COMT* genes

**Figure 4:**
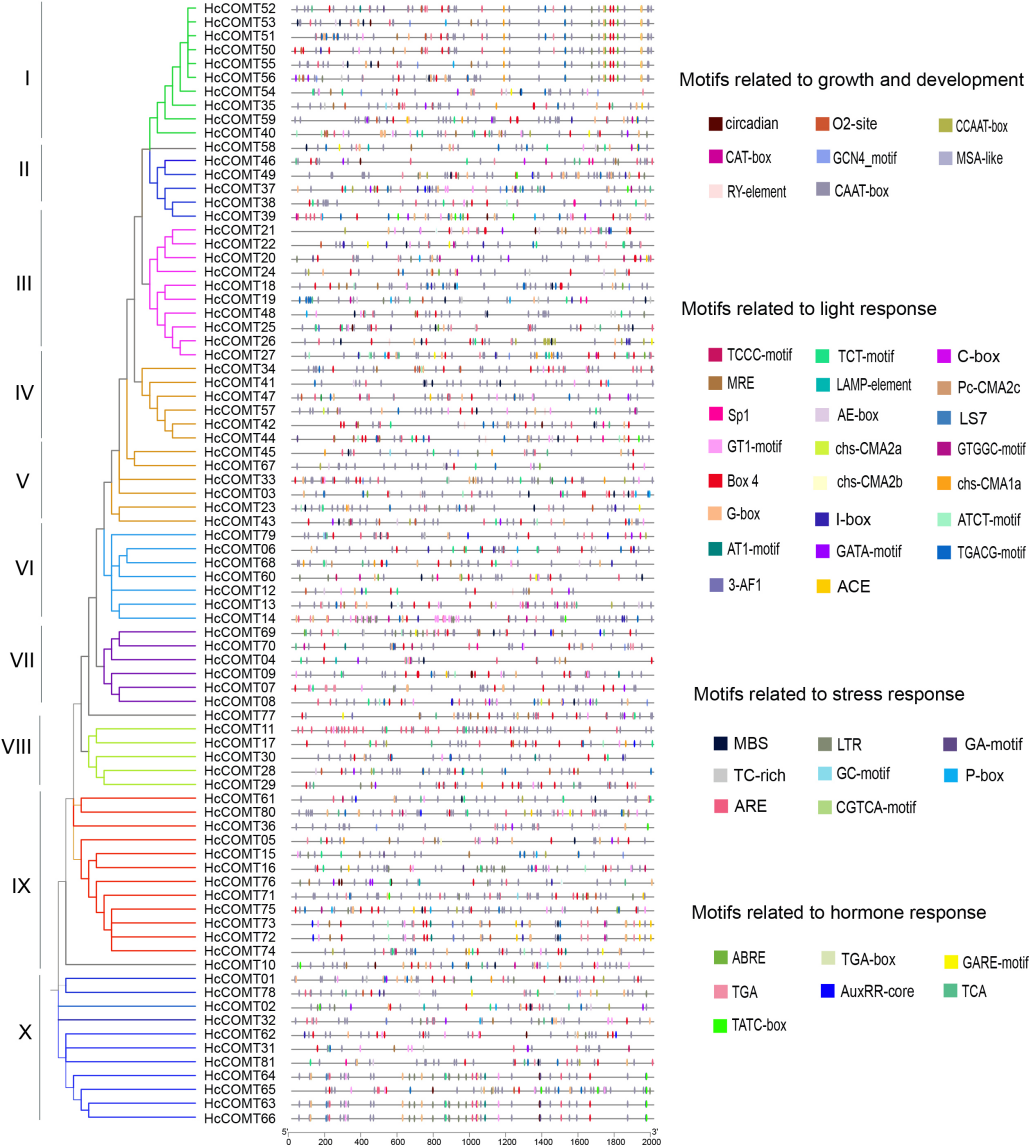
Cis-elements in promoters for *HcCOMT* genes. A total of 4 classes cis-elements related growth and development, light response, stress response and hormone response were found in the 2000 bp upstream promoters of *HcCOMT* genes. The different colors indicate different cis-acting elements.

To explore the transcriptional regulation of the *HcCOMT* members, the 2-kb region upstream of each translation start codon was scanned with PlantCARE. In total, 81 promoters harbored various enriched elements that could be assigned to four functional categories: (i) plant growth and development elements, (ii) light response elements, (iii) abiotic and biotic stress response elements, and (iv) plant hormone response elements (**Figure 4**).

Light-responsive elements including Box4, G-box, GT1-motif, MRE and ACE, were the most abundant class, present in every *HcCOMT* gene promoter and suggesting ubiquitous light regulation. Hormone-responsive elements were also widespread: ABRE (abscisic acid), AuxRE (auxin), GARE (gibberellin) and TCA/SA-responsive elements (salicylic acid) were identified in *HcCOMT78*, *HcCOMT65*, *HcCOMT59* and *HcCOMT71* promoters, respectively. Notably, *HcCOMT03* contained six ABRE elements, whereas *HcCOMT05* carried seven TCA elements, implying their potential roles in ABA- and SA-mediated stress responses. Stress-related elements such as MBS (MYB-binding site, drought-inducible), LTR (low-temperature responsive) and TC-rich repeats (defence and stress) were enriched in 61 promoters. Development-associated elements (CAAT-box, CAT-box, CCAAT-box, O2-site and GCN4-motif) were particularly prominent in *HcCOMT19*, which harbored five distinct growth-related elements (**Figure 4**). Collectively, the diversity and multiplicity of these cis-elements indicate that *HcCOMT* genes are subject to combinatorial control by light, hormonal and environmental cues.

### 3.6 GO functional enrichment analysis of the *HcCOMT* genes

**Figure 5:**
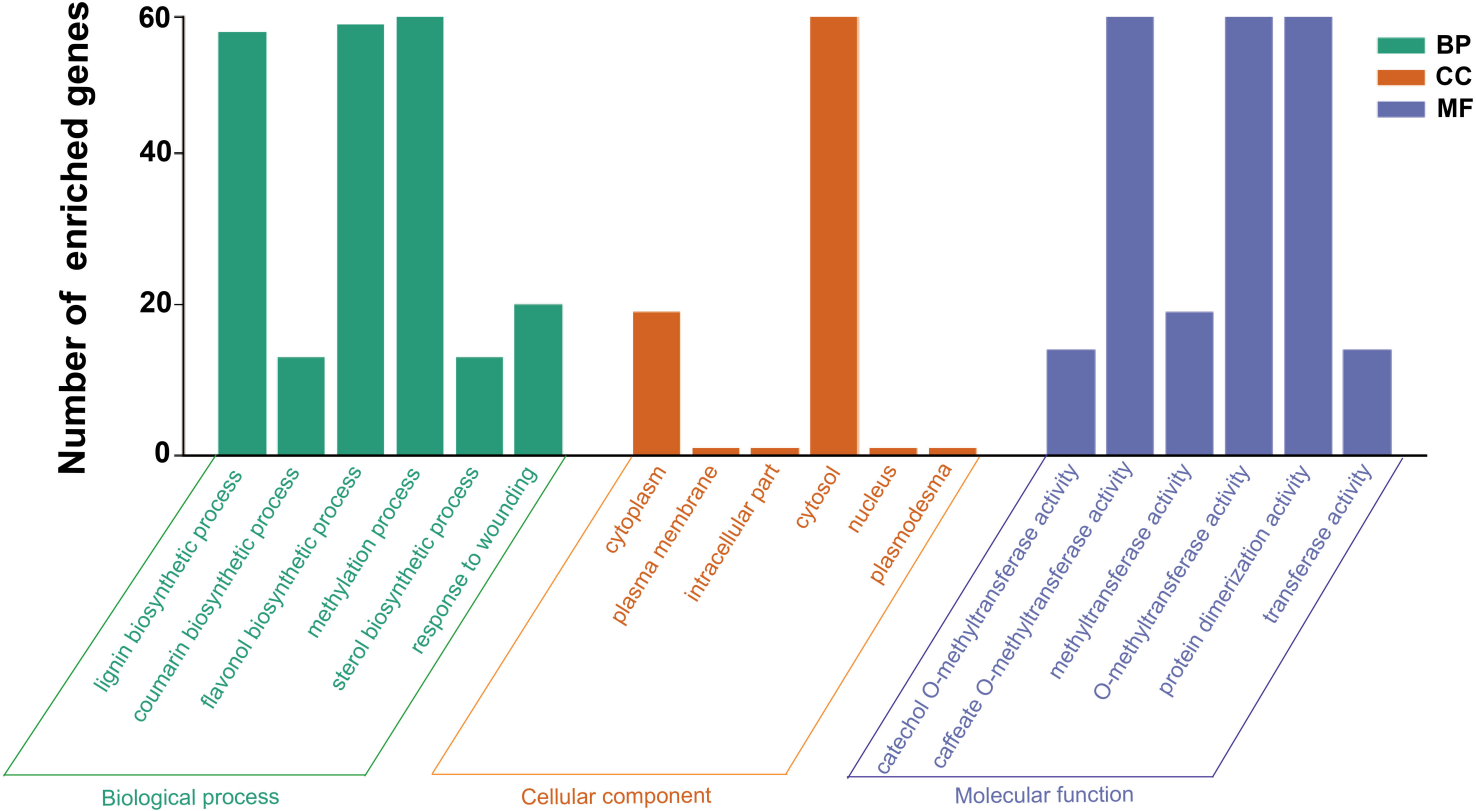
The potential function enrichment analysis of *HcCOMT* genes in kenaf.

To further elucidate the biological functions of the *HcCOMT* gene family, we conducted Gene Ontology (GO) enrichment analysis of the 81 *HcCOMT* genes. As shown in **Figure 5**, the members of the COMT family of kenaf are mainly enriched in the biosynthetic process, cellular process, and transferase activity processes. Within the “biological processes” ontology, “lignin biosynthetic process” (GO:0009808), “flavanol biosynthetic process” (GO:0051555) and “methylation process” (GO:0032259) were the most enriched terms. Cellular component analysis placed the majority of proteins in the cytoplasm (GO:000537) and cytosol activity (GO:0005829). Under “molecular function”, caffeate O-methyltransferase activity (GO:0047761), O-methyltransferase activity (GO:0008171), protein dimerization activity (GO:0046983), and general methyltransferase activity (GO:0008168) were significantly enriched, consistent with the catalytic and oligomeric properties of COMT enzymes.

### 3.7 Structural divergence of salt-responsive HcCOMT proteins

**Figure 6:**
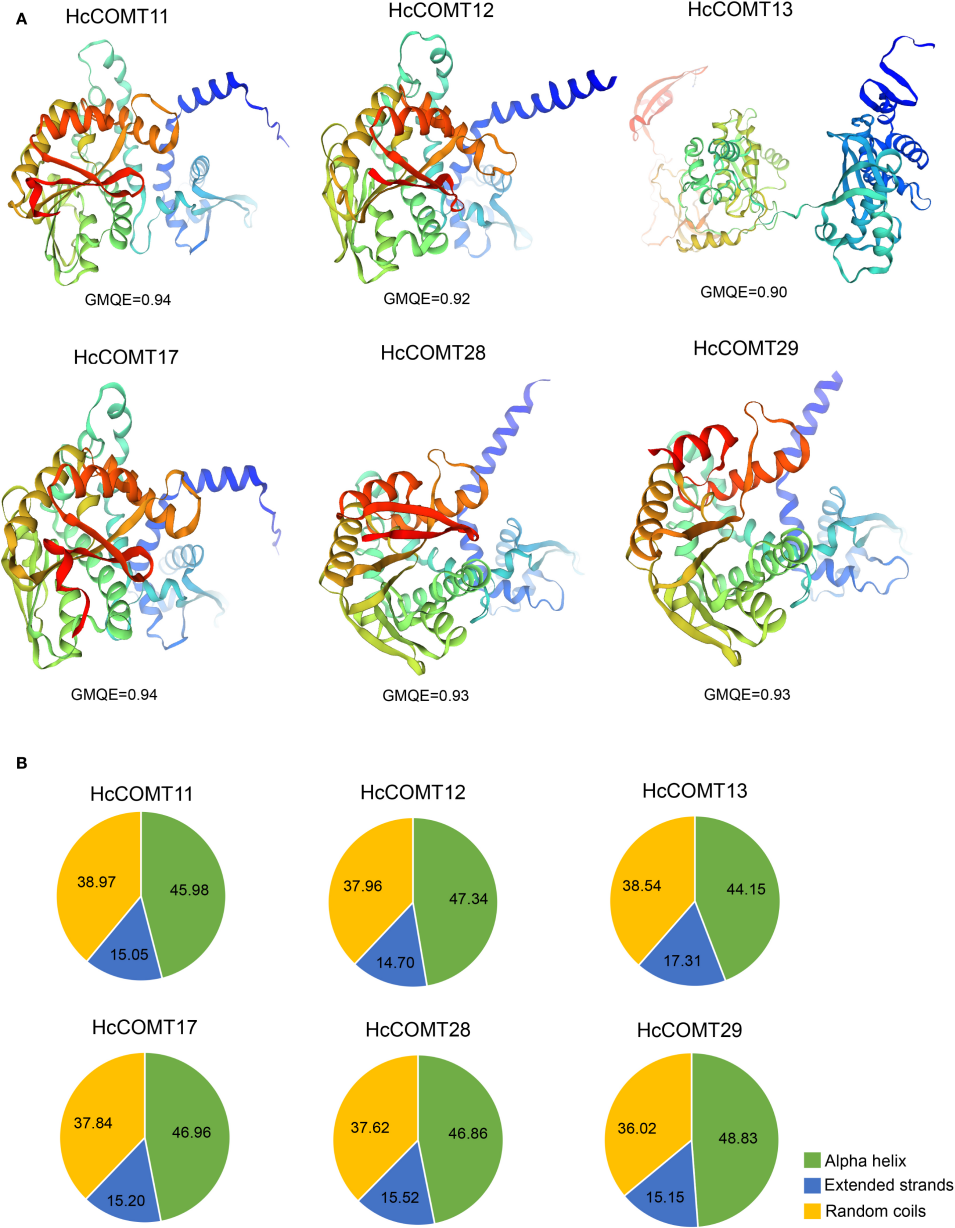
Secondary structure prediction of *HcCOMT* proteins. **(A)** 3-D modelling of the secondary structure in each group of six *HcCOMT* proteins. **(B)** Average ratios of alpha helix, extended strand, and random coil structures of six *HcCOMT* proteins in each cluster.

Six HcCOMT genes (*HcCOMT11*, *HcCOMT12*, *HcCOMT13*, *HcCOMT17*, *HcCOMT28* and *HcCOMT29*) that exhibited significant transcriptional modulation under salinity stress were selected for structural modeling. High-quality 3-D models (GMQE ≥ 0.75; QMEAN ≥ -1.5) were generated with SWISS-MODEL platform. Spatial superposition revealed marked conformational divergence among the proteins (**Figure 6A**). HcCOMT12, HcCOMT13, HcCOMT28, and HcCOMT29 proteins adopted distinct folds, whereas HcCOMT11 and HcCOMT17 proteins displayed nearly identical architectures, suggesting that the latter pair may represent functionally redundant isoforms (**Figure 6A**).

The 3D structural diversity is mainly relied on their secondary structure composition. To explore their structural diversity, we conducted a secondary structure prediction analysis for these proteins. The results revealed that six HcCOMT proteins are dominated by alpha helices (44.15% - 48.83%), followed by random coils (36.02% - 38.97%), and extended strands (14.70% - 17.31%) (**Figure 6B**). On average, alpha helices constitute 46.69% of the residues, whereas extended strands contribute only 15.49% (**Figure 6B**). The high alpha helical content corresponds to the N-terminal dimerization domain, whereas the catalytic methyltransf-2 domain is embedded within mixed α/β regions, consistent with the canonical fold of plant O-methyltransferases. The observed structural variability may underpin differential substrate specificity or interaction networks under salt stress.

### 3.8 Protein-protein interaction landscape of salt-responsive HcCOMT proteins

**Figure 7:**
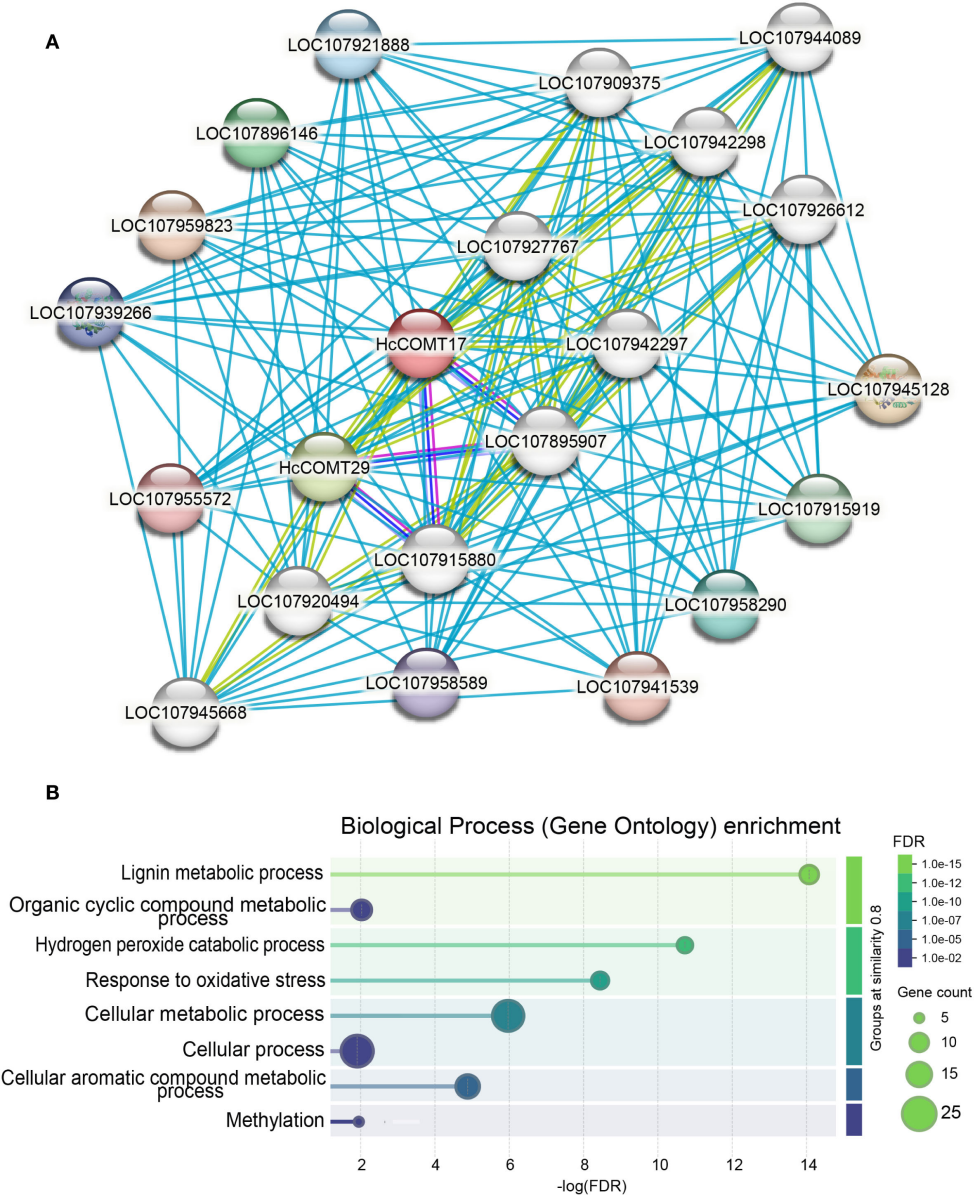
Co-expression and functional prediction analysis of six *HcCOMT* proteins. **(A)** Co-expression network of *HcCOMTs*. The correlation coefficients are shown with different colours in node genes. Light gray lines indicate the interaction links between node *HcCOMTs* and other genes. **(B)** GO enrichment analysis of co-expressed *HcCOMT* genes.

To contextualize the functional roles of the six salt-responsive HcCOMT proteins, we interrogated their orthologous in *Gossypium hirsutum* using the STRING protein interaction Database. A confidence score threshold of 0.9 was applied to ensure reliable protein-protein interaction predictions. The resulting network resolved into two densely connected modules that converge on lignin biosynthesis and oxidative stress mitigation (**Figure 7A**, **Supplementary Table S5**). Module 1 is associated with lignin polymerization hub. For example, HcCOMT17 and HcCOMT29 (their orthologous in cotton) directly interact with five class III peroxidase 10 (LOC107945128), 24 (LOC107896146), 27 (LOC107915919), 29 (LOC107958290), and 31 (LOC107921888), which is involved in the final oxidative coupling of monolignols, and with four UDP-glycosyltransferase of the 72E clade (LOC107909375, LOC107942297, LOC107920494, and LOC107942298), which convert cinnamoyl-CoA esters to cinnamaldehydes. Together, these enzymes constitute a contiguous metabolic channel that links O-methylation to cell-wall lignification. Module 2 is involved into the phenylpropanoid branch-point and antioxidant coupling. HcCOMT29 forms reciprocal interactions with caffeic acid 3-O-methyltransferase (LOC107915880 and LOC107895907) and anthocyanidin 3-O-glucosyltransferase 5 (UFGT5) (LOC107927767, LOC107945668, LOC107944089, and LOC107926612). The former regulates the methylation of lignin precursors, whereas the latter diverts phenylpropanoid flux towards anthocyanin biosynthesis, thereby modulating both lignin quantity and antioxidant capacity. Importantly, the peroxidase cluster also interfaces with hydrogen peroxide detoxification pathways, providing direct crosstalk between lignin synthesis and oxidative stress signaling.

To further elucidate the biological process of those interaction proteins, we conducted GO enrichment analysis of these interacting proteins. As shown in **Figure 7B**, GO enrichment confirmed that the network is significantly enriched for “lignin metabolic process”, “hydrogen peroxide catabolic process”, and “response to oxidative stress processes” (**Figure 7B**). Collectively, these data position the HcCOMT17 and HcCOMT29 proteins at a metabolic nexus that integrates lignin deposition with redox homeostasis under salt stress.

### 3.9 Spatial and temporal expression pattern of *HcCOMT* genes under salt stress

**Figure 8:**
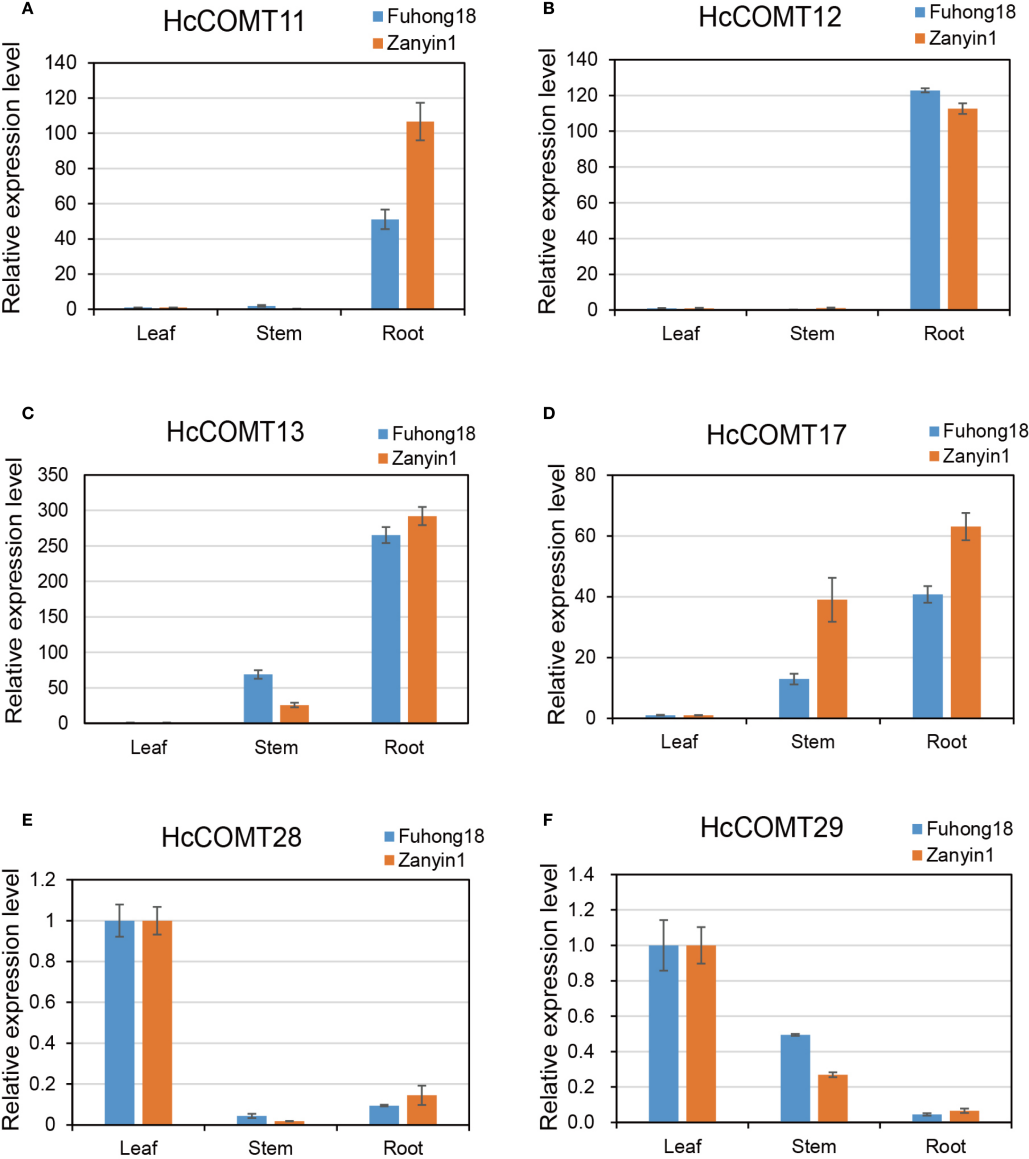
Expression profiles for *HcCOMT* genes in different tissues. The expression profiles of six represented *HcCOMT* genes across root stem and leaf in kenaf: *HcCOMT11* **(A)**, *HcCOMT12* **(B)**, *HcCOMT13* **(C)**, *HcCOMT17* **(D)**, *HcCOMT28* **(E)** and *HcCOMT29* **(F)** based on the qRT-PCR analysis.

Firstly, transcript abundance of six candidate HcCOMT genes was surveyed in root, stem and leaf tissues of the salt-tolerant cultivar Fuhong18 and the salt-sensitive cultivar Zanyin1 under control conditions. As depicted in **Figure 8**, the transcription abundance of these *HcCOMT* genes varied across the different tissue samples. *HcCOMT11* and *HcCOMT12* were predominantly expressed in roots (**Figures 8A, B**), whereas *HcCOMT13* and *HcCOMT17* accumulated to the highest levels in both roots and stems (**Figure 8C, D**). In contrast, *HcCOMT28* and *HcCOMT29* exhibited leaf-restricted expression (**Figures 8E, F**). Notably, *HcCOMT11* and *HcCOMT12* displayed nearly identical organ-specific patterns, which may suggest possible redundancy or co-regulation.

**Figure 9:**
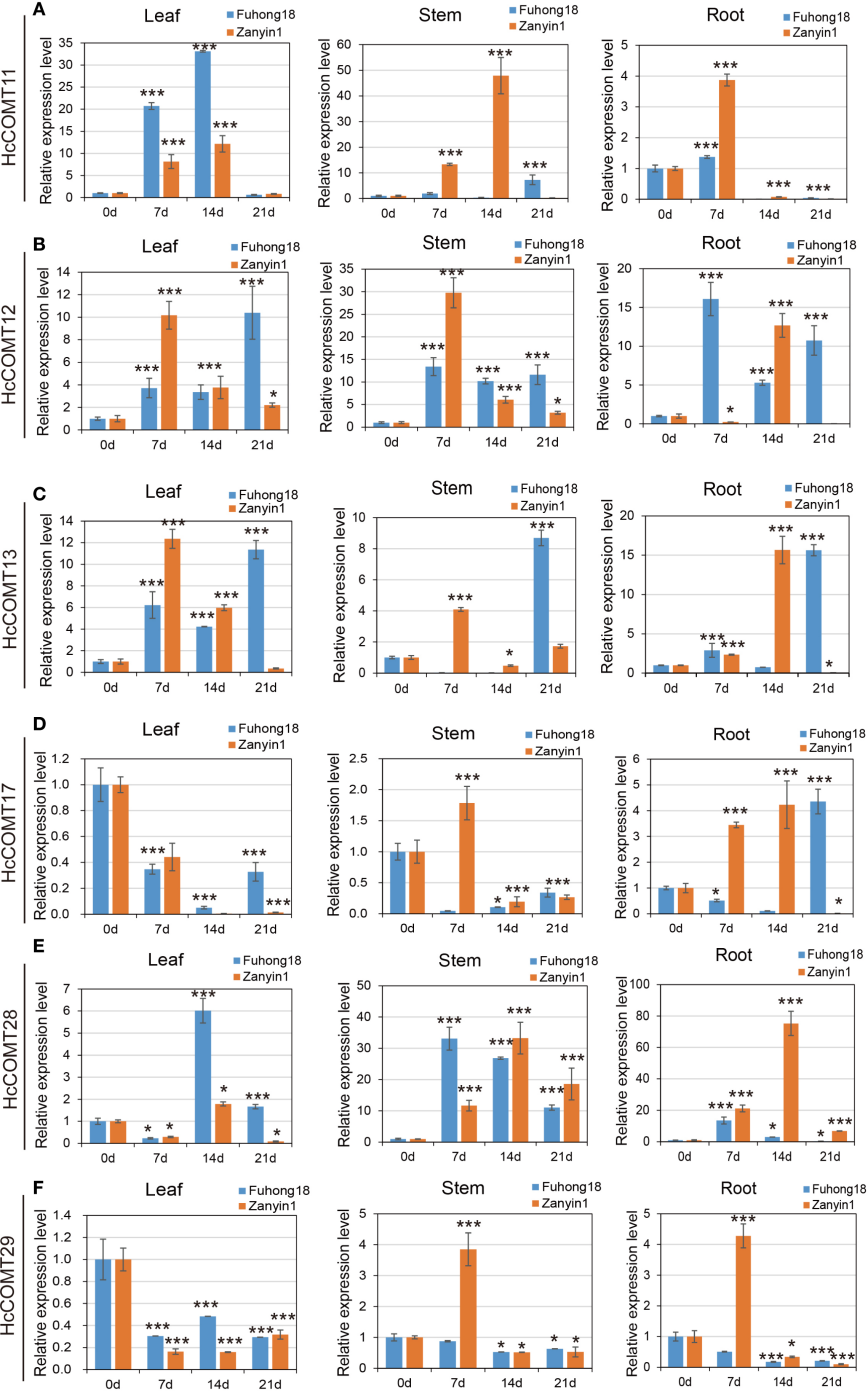
Relative expression level of *HcCOMT* genes, *HcCOMT11* **(A)**, *HcCOMT12* **(B)**, *HcCOMT13* **(C)**, *HcCOMT17* **(D)**, *HcCOMT28* **(E)**, and *HcCOMT29* **(F)**, in different tissues of kenaf under salinity stress conditions. Plants with regular water were assigned as control group. The relative gene expression levels were normalized against the internal reference gene (PP2A). Data represent the mean ± standard error of three independent biological replicates, each with three technical replicates. Statistical significance was determined using one-way ANOVA followed by Tukey’s HSD test. * and *** above the bars indicate differences between treatments at *p* ≤ 0.05 and *p* ≤ 0.01, respectively.

Then, quantitative RT-PCR was used to monitor the temporal response of the six *HcCOMT* genes to 200 mM NaCl over 21 days. Data are presented as fold change relative to the 0-day control for each tissue and cultivar (**Figure 9**). For *HcCOMT11* gene in salt-sensitive cultivar Zanyin1, maximal induction in stems at 14 d (48-fold), moderate accumulation in leaves, and negligible expression in roots (**Figure 9A**); while in salt-tolerant cultivar Fuhong18, *HcCOMT11* was upregulated strongly in the leaves (32-fold at 14 d), with transcripts almost undetectable in roots and stems. For *HcCOMT12* gene in cultivar Fuhong18, sustained high expression across all organs, and in cultivar Zanyin1, *HcCOMT12* gene was transient root-specific peak at 14 d (12-fold), followed by smaller increased in leaves and stems at 7 d (**Figure 7B**). For HcCOMT13 gene, both cultivars exhibited highest transcript levels in leaves at 7 d and 14 d. In Fuhong18, the expression further increased in stems and roots at 21 d, indicating a coordinated systemic response where absent in Zanyin1 (**Figure 9C**). *HcCOMT17* displayed significantly down-regulation relative to controls in the leaves of both cultivars (p < 0.01). Zanyin1 showed transient stem induction at 7 d and a root-specific peak at 14 d, whereas Fuhong18 exhibited delayed root activation at 21 d (**Figure 9D**). *HcCOMT28* showed a progressive up-regulation in stems of both cultivars, and peaking at 14 d (25-fold in stem of Fuhong18, 32-fold in Zanyin1). Fuhong18 additionally displayed leaf induction at 14 d (6-fold) and root repression after 7 days treatments (**Figure 9E**). *HcCOMT29* exhibited continuous down-regulation in the leaves and stems of both cultivars. In Zanyin1, a transient increase was observed in stems and roots at 7 d (3.8-fold and 4.2-fold, respectively), whereas Fuhong18 maintained low and stable levels (**Figure 9F**).

Collectively, the six *HcCOMT* genes exhibit cultivar-and tissues-specific transcriptional programmers that align with their predicted physiological roles: root/stem isoforms (*HcCOMT11*, *HcCOMT12*, *HcCOMT13*, and *HcCOMT17*) are preferentially induced in the tolerant cultivar, whereas leaf-specific members (*HcCOMT28* and *HcCOMT29*) are repressed, reflecting a lignin deposition strategy that may contribute to the superior salt tolerance of Fuhong18.

## 4 Discussion

The secondary cell wall acts as a front-line barrier against both biotic and abiotic stresses, and lignin is one of its most critical hydrophobic constituents. By stiffening the wall and limiting apoplastic water movement, *COMT* regulates the syringyl-to-guaiacyl (S/G) ratio in lignin, a determinant of both cell wall rigidity and adaptability under abiotic stress (Vanholme et al., 2010; Lv et al., 2011; Oraby and Ramadan, 2014; Tuskan et al., 2019). This dual role makes *COMT* an attractive breeding target for enhancing salt tolerance and improving fiber quality simultaneously. In saline environments, higher S-lignin deposition can strengthen secondary walls, restrict Na^+^ influx, and stabilize cell structure under osmotic stress, thereby improving resilience. At the same time, fine-tuning lignin composition can optimize fiber flexibility, strength, and industrial processing traits. Similar strategies have already been demonstrated in other fiber and forage crops, highlighting the translational potential of our findings for kenaf. In *Populus*, manipulation of lignin biosynthetic genes including *COMT* altered the S/G ratio and enhanced salt tolerance by reducing ion penetration through reinforced secondary cell walls (Janz et al., 2012; He et al., 2013). In forage legumes such as *Medicago sativa*, transcriptomic and functional studies revealed that *COMT* and other monolignol pathway genes contribute not only to lignin deposition under salt and drought stress but also to forage digestibility and fiber strength (Rahman et al., 2022).

Here, we provide the first genome-wide catalogue of the kenaf (*Hibiscus cannabinus* L.) caffeoyl acid 3-O-methyltransferase (COMT) gene family, the key methyltransferase that channels precursors toward syringyl (S) lignin units, and demonstrate that specific members are transcriptionally reprogrammed during salt stress. A total of 81 *HcCOMT* genes were resolved, which can be grouped into ten clades (Group I-X) that largely mirror the phylogenetic structure of *Arabidopsis* COMTs. Notably, *HcCOMT81*, the kenaf orthologue of *Arabidopsis* At5G54160 (*COMT01*), shares 98% amino acid identity with *AtCOMT01*. Previous work has shown that loss of *AtCOMT01* abolishes S-lignin units and causes accumulation of the atypical monomer caffeic alcohol, whereas over-expression enhances melatonin synthesis and salt tolerance (Lee et al., 2014; Eudes et al., 2017; Yang et al., 2019). The tight ortholog therefore predicts a conserved role for *HcCOMT81* in both lignification and stress-responsive melatonin metabolism (Eudes et al., 2017).

Duplication events can explain the amplification and loss of genes during the evolution of species, and gene duplication may also contribute to the evolution of species and help plants to adapt to their surrounding growth environment (Bhardwaj et al., 2005; Wang et al., 2024). Intraspecific and interspecific synteny analyses indicate that the kenaf *COMT* gene family has expanded mainly through segmental duplication; only 14 fragmentally duplicated pairs and 3 tandem clusters were detected among the 81 loci. All retained duplicates exhibited Ka/Ks less than 1, signifying strong purifying selection. The scarcity of COMT collinear blocks between kenaf and Arabidopsis (only 2 pairs) further underscores the ancient and conserved nature of this family, consistent with patterns reported in rice (Liang et al., 2022), blueberry (Liu et al., 2021), and cotton (Wu et al., 2021).

Interaction networks generated via STRING place kenaf *HcCOMT11* and *HcCOMT12* at the core of the lignin polymerization machinery. Their orthologous interact physically with cinnamoyl-CoA reductase (IRX4), which is involved in the later stages of lignin biosynthesis, converting cinnamoyl coenzyme A to the corresponding cinnamaldehyde (Abdelaziz et al., 2016; Wu et al., 2021). *HcCOMT10*, *HcCOMT16*, *HcCOMT27*, and *HcCOMT28* belong to the homologous proteins with *OMT1* and are related to *CYP84A1*, which belongs to the ferulic acid 5-hydroxylase and encodes ferulic acid 5-hydroxylase (F5H), involving in lignin biosynthesis and multiple peroxidases, forming a metabolic channel that converts phenylpropanoid acids into lignin monomers while simultaneously regulating H_2_O_2_ homeostasis (Meyer et al., 1998; Vanholme et al., 2019). These data corroborate the dual role of COMT enzymes in cell-wall fortification and redox balance. The phylogenetic tree showed that *HcCOMT81* and *OMT1* belonged to the same branch, and it was hypothesized that there might be a similar gene function between the two, in which *OMT1* catalyzes the methylation of lignin precursors, which participates in melatonin biosynthesis (Do et al., 2007), and is involved in the growth and development of the plant, the biotic and abiotic stress responses, and regulates plant response to environmental stresses such as drought and salt damage (Moura et al., 2010; Liu et al., 2018; Yang et al., 2019).

Given that roots are the earliest site to perceive salt stress. Tissue-specific expression profiling showed that *HcCOMT11*, *HcCOMT12*, *HcCOMT13*, and *HcCOMT17* are predominantly expressed in roots and stems, the organs that initially perceive and transduce saline signals. Exposure to 200 mM NaCl elicited cultivar-dependent induction kinetics: transcript levels of these four genes rose earlier and accumulated to higher abundance in the salt-tolerant cultivar Fuhong18, mirroring the enhanced lignin deposition observed in its stems. In contrast, the leaf-specific isoforms *HcCOMT28* and *HcCOMT29* were largely repressed, implying a resource reallocation from photosynthetic tissues to the vasculature under stress. The coordinated up-regulation of root/stem COMTs coupled with the down-regulation of leaf COMTs provides a mechanistic explanation for the superior lodging resistance and Na^+^ exclusion capacity of Fuhong18. To obtain a comprehensive understanding of kenaf salinity responses, future work should encompass a broader NaCl concentration gradient and employ functional assays (such as overexpression or CRISPR/Cas9-mediated knockout in kenaf or heterologous systems) to directly establish the contribution of these *HcCOMTs* to lignin modification and salt stress adaptation.

## 5 Conclusion

A total of 81 *HcCOMT* genes (*HcCOMT01*-*HcCOMT81*) were identified in this study, distributed on 15 chromosomes. Phylogenetic reconstruction delineated ten well-supported clades I-X, with kenaf orthologous clustering in clades III, VIII and X. qPCR analyses revealed that six stress-responsive members, particularly *HcCOMT11*, *HcCOMT12*, *HcCOMT13* and *HcCOMT17*, are specifically upregulated in stems under salt stress and correlate positively with lignin accumulation. These findings provide a robust genomic resource and a functional framework for dissecting the molecular basis of lignin-mediated salt tolerance in kenaf, and lay the groundwork for marker-assisted selection or genome editing of COMT loci to enhance fiber quality and abiotic stress resilience.

## Data Availability

The original contributions presented in the study are included in the article/**Supplementary Material**. Further inquiries can be directed to the corresponding authors.
