# Supplementary material for: Comprehensive analysis of the Caffeic acid O-methyltransferase gene family in kenaf (Hibiscus cannabinus L.) and their expression characteristics in response to salinity stress
Source: Front Plant Sci. 2025 Oct 7;16:1678383. doi: 10.3389/fpls.2025.1678383 (PMC12537682; doi:10.3389/fpls.2025.1678383)
Supplement: Supplementary file 1 [file DataSheet1.docx]

**Comprehensive analysis of the Caffeic acid O-methyltransferase (COMT) gene family in kenaf (*Hibiscus cannabinus* L.) and their expression characteristics in response to salinity stress**

Jiantang Xu^1,#^, Tianjin Liu^1,#^, Hui Lin^1,2^, Rong Huang^1^, Meixia Chen^1,2^, Pingping Fang^1^*, Xiaoping Niu^1^*

^1^ Key Lab of Genetics, Breeding and Multiple Utilization of Crops, Ministry of Education, Fujian Provincial Key Laboratory of Haixia Applied Plant Systems Biology, College of Life Science, College of Agriculture, Fujian Agriculture and Forestry University, Fuzhou 350002, China;

^2^ College of Life Science, Industry and University Research Cooperation Demonstration Base in Fujian Province, Ningde Normal University, Ningde, China

^#^ These authors contributed to this article equally.

* Corresponding authors. E-mail: xpniu0613@126.com, f3@fafu.edu.cn

**Supplemental Figure 1. Chromosome distributions of *HcCOMTs*.** All 81 *COMT* genes in the kenaf are marked in 25 pairs of chromosomes. Only 15 chromosomes contain the *COMT* gene, and several chromosomes show a dense distribution for *COMT* genes in specific segments, which implies the gene duplication events in the kenaf genome.


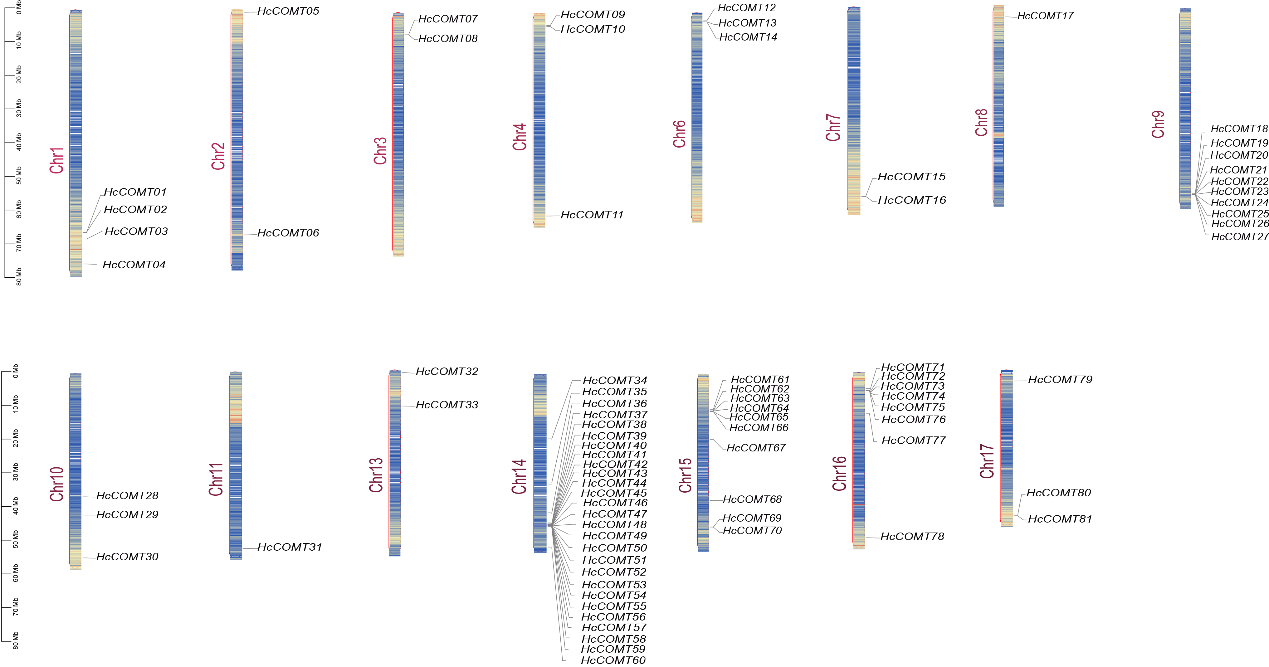


**Supplemental Figure 2. Primer specificity validation for six *HcCOMT* genes.** RT-PCR was performed with gene-specific primers for each of the 6 HcCOMT members. Amplicons were separated on a 1% agarose gel; single bands of the expected size and the absence of non-specific products confirm primer specificity. Marker: DNA Marker A.


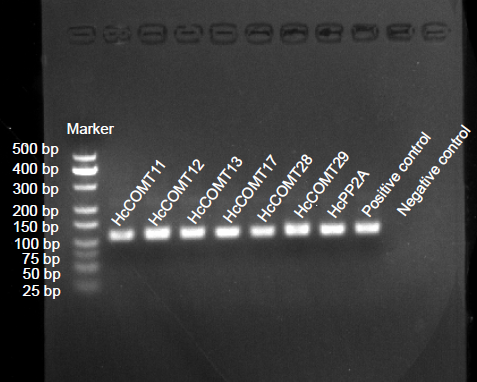


**Supplemental Tables**

**Table S1. Primer sequences used for qRT-PCR analysis.**

| **Target genes** | **Forward primer** | **Reverse primer** |
| --- | --- | --- |
| *HcCOMT11* | GGAGCATGTAGGAGGAGATA | CCCAATCGTGAAGTACACTC |
| *HcCOMT12* | GGGACTGCTTTGAGTATGTTAG | CTTTCGATGCCGTCGTATTT |
| *HcCOMT13* | CATAATACCGCCGATGCAC | GTCGTCCCAATCATGCAAA |
| *HcCOMT17* | ACATGTTCGAGGTGGTTTC | GACTTAAGCCCTCAAATCCC |
| *HcCOMT28* | GGCCACCGATGATGAAAT | CTGCAAACCCAGTGTAAGT |
| *HcCOMT29* | CCTCATCCTCCCTCAGATAATA | CCGAAAGGAAGAGACGAAAG |

**Table S2. The COMT genes of *Arabidopsis thaliana* used for phylogenetic analysis.**

| **Species** | **Gene name** | **Gene ID** |
| --- | --- | --- |
| **Arabidopsis thaliana** | AtCOMT1 | AT1G21100 |
|  | AtCOMT2 | AT1G21110 |
|  | AtCOMT3 | AT1G21120 |
|  | AtCOMT4 | AT1G21130 |
|  | AtCOMT5 | AT1G33030 |
|  | AtCOMT6 | AT1G51990 |
|  | AtCOMT7 | AT1G63140 |
|  | AtCOMT8 | AT1G76790 |
|  | AtCOMT9 | AT1G77520 |
|  | AtCOMT10 | AT1G77530 |
|  | AtCOMT11 | AT3G53140 |
|  | AtCOMT12 | AT4G35150 |
|  | AtCOMT13 | AT4G35160 |
|  | AtCOMT14 | AT5G37170 |
|  | AtCOMT15 | AT5G53810 |
|  | AtCOMT16 | AT5G54160 |

**Table S3. The selection pressure (Ka/Ks) of COMT genes in *H. cannabinus*.**

| **Seq_1** | **Seq_2** | **Ka** | **Ks** | **Ka/Ks** |
| --- | --- | --- | --- | --- |
| HcCOMT16 | HcCOMT75 | 0.160610991 | 1.000727787 | 0.160494186 |
| HcCOMT07 | HcCOMT09 | 0.051199318 | 0.366414741 | 0.139730508 |
| HcCOMT23 | HcCOMT38 | 0.435419221 | 0.936911076 | 0.464739112 |
| HcCOMT12 | HcCOMT13 | 0.267266553 | 0.804555149 | 0.332191713 |
| HcCOMT04 | HcCOMT69 | 0.080740395 | 0.453471876 | 0.178049399 |
| HcCOMT17 | HcCOMT29 | 0.31192457 | 1.778683473 | 0.175368229 |
| HcCOMT12 | HcCOMT79 | 0.178169815 | 0.618371141 | 0.288127637 |
| HcCOMT31 | HcCOMT65 | 0.037048317 | 0.732725384 | 0.05056235 |
| HcCOMT31 | HcCOMT80 | 0.394495868 | 4.206104179 | 0.093791274 |
| HcCOMT62 | HcCOMT81 | 0.065258533 | 0.45686197 | 0.142840808 |

**Table S4. The COMT-related locus in synteny analysis among *Arabidopsis*, cotton, and kenaf.**

| **Species** | **Chromosome** | **Gene Name** |
| --- | --- | --- |
| Gossypium hirsutum | Ghir_D13/Hc-Chr08 | GhCOMT56/HcCOMT17 |
|  | Ghir_D13/Hc-Chr10 | GhCOMT56/HcCOMT29 |
|  | Ghir_D12/Hc-Chr02 | GhCOMT52/HcCOMT05 |
|  | Ghir_D12/Hc-Chr06 | GhCOMT50/HcCOMT12 |
|  | Ghir_D12/Hc-Chr11 | GhCOMT53/HcCOMT31 |
|  | Ghir_D12/Hc-Chr17 | GhCOMT53/HcCOMT80 |
|  | Ghir_A10/Hc-Chr09 | GhCOMT21/HcCOMT18 |
|  | Ghir_A10/Hc-Chr14 | GhCOMT20/HcCOMT55 |
|  | Ghir_A10/Hc-Chr14 | GhCOMT21/HcCOMT58 |
|  | Ghir_A10/Hc-Chr14 | GhCOMT20/HcCOMT37 |
|  | Ghir_A10/Hc-Chr14 | GhCOMT21/HcCOMT40 |
|  | Ghir_A10/Hc-Chr14 | GhCOMT18/HcCOMT36 |
|  | Ghir_A13/Hc-Chr08 | GhCOMT29/HcCOMT17 |
|  | Ghir_A13/Hc-Chr10 | GhCOMT29/HcCOMT29 |
|  | Ghir_D10/Hc-Chr09 | GhCOMT47/HcCOMT24 |
|  | Ghir_D10/Hc-Chr14 | GhCOMT46/HcCOMT55 |
|  | Ghir_D10/Hc-Chr14 | GhCOMT47/HcCOMT56 |
|  | Ghir_D10/Hc-Chr14 | GhCOMT46/HcCOMT37 |
|  | Ghir_D10/Hc-Chr14 | GhCOMT49/HcCOMT40 |
|  | Ghir_D10/Hc-Chr14 | GhCOMT43/HcCOMT36 |
|  | Ghir_A12/Hc-Chr02 | GhCOMT25/HcCOMT05 |
|  | Ghir_A12/Hc-Chr02 | GhCOMT27/HcCOMT05 |
|  | Ghir_A12/Hc-Chr06 | GhCOMT23/HcCOMT12 |
|  | Ghir_A12/Hc-Chr17 | GhCOMT27/HcCOMT80 |
|  | Ghir_D09/Hc-Chr07 | GhCOMT41/HcCOMT15 |
|  | Ghir_D09/Hc-Chr16 | GhCOMT41/HcCOMT75 |
|  | Ghir_A06/Hc-Chr01 | GhCOMT11/HcCOMT03 |
|  | Ghir_D04/Hc-Chr06 | GhCOMT33/HcCOMT13 |
|  | Ghir_D04/Hc-Chr06 | GhCOMT33/HcCOMT12 |
|  | Ghir_D04/Hc-Chr17 | GhCOMT33/HcCOMT79 |
|  | Ghir_A04/Hc-Chr06 | GhCOMT06/HcCOMT13 |
|  | Ghir_A04/Hc-Chr06 | GhCOMT06/HcCOMT12 |
|  | Ghir_A04/Hc-Chr17 | GhCOMT06/HcCOMT79 |
|  | Ghir_D02/Hc-Chr03 | GhCOMT31/HcCOMT07 |
|  | Ghir_D02/Hc-Chr04 | GhCOMT31/HcCOMT09 |
|  | Ghir_A05/Hc-Chr07 | GhCOMT10/HcCOMT15 |
|  | Ghir_A05/Hc-Chr16 | GhCOMT10/HcCOMT75 |
|  | Ghir_D08/Hc-Chr11 | GhCOMT39/HcCOMT31 |
|  | Ghir_D08/Hc-Chr15 | GhCOMT39/HcCOMT62 |
|  | Ghir_D08/Hc-Chr17 | GhCOMT39/HcCOMT81 |
|  | Ghir_A08/Hc-Chr08 | GhCOMT14/HcCOMT17 |
|  | Ghir_A08/Hc-Chr10 | GhCOMT14/HcCOMT29 |
|  | Ghir_A08/Hc-Chr11 | GhCOMT13/HcCOMT31 |
|  | Ghir_A08/Hc-Chr15 | GhCOMT13/HcCOMT62 |
|  | Ghir_A08/Hc-Chr17 | GhCOMT13/HcCOMT81 |
|  | Ghir_A09/Hc-Chr07 | GhCOMT16/HcCOMT15 |
|  | Ghir_A09/Hc-Chr16 | GhCOMT16/HcCOMT75 |
|  | Ghir_A03/Hc-Chr03 | GhCOMT03/HcCOMT07 |
|  | Ghir_A03/Hc-Chr04 | GhCOMT03/HcCOMT09 |
| Arabidopsis thaliana | At-Chr05/Hc-Chr17 | AtCOMT15/HcCOMT81 |
|  | At-Chr01/Hc-Chr17 | AtCOMT05/HcCOMT80 |

**Table S5. Information of HcCOMTs predicted interaction proteins.**

| **Gene** | **Locus ID** | **Homologs Definition Predicted** |
| --- | --- | --- |
| LOC107945128 | XM_016878986 | Gossypium hirsutum peroxidase 10 |
| LOC107896146 | XM_016821269 | Gossypium hirsutum peroxidase 24 |
| LOC107915919 | XM_016845109 | Gossypium hirsutum peroxidase 27 |
| LOC107958290 | XM_041113664 | Gossypium hirsutum peroxidase 29 |
| LOC107921888 | XM_016851710 | Gossypium hirsutum peroxidase 31 |
| LOC107959823 | XM_016895979 | Gossypium hirsutum peroxidase 31 |
| LOC107939266 | XM_016872569 | Gossypium hirsutum peroxidase 31 |
| LOC107955572 | XM_016891363 | Gossypium hirsutum peroxidase 31 |
| LOC107941539 | XM_016875084 | Gossypium hirsutum peroxidase 31 |
| LOC107958589 | NM_001327676 | Gossypium hirsutum peroxidase 31-like |
| LOC107909375 | XM_016836864 | Gossypium hirsutum UDP-glycosyltransferase 72E1 |
| LOC107942297 | XM_016875931 | Gossypium hirsutum UDP-glycosyltransferase 72E1 |
| LOC107920494 | XM_016850236 | Gossypium hirsutum UDP-glycosyltransferase 72E1 |
| LOC107942298 | XM_016875947 | Gossypium hirsutum UDP-glycosyltransferase 72E2 |
| LOC107915880 | XM_016845074 | Gossypium hirsutum caffeic acid 3-O-methyltransferase |
| LOC107895907 | XM_016821118 | Gossypium hirsutum caffeic acid 3-O-methyltransferase |
| LOC107927767 | XM_016858876 | Gossypium hirsutum anthocyanidin 3-O-glucosyltransferase 5 |
| LOC107945668 | XM_016879757 | Gossypium hirsutum anthocyanidin 3-O-glucosyltransferase 5 |
| LOC107944089 | XM_016877906 | Gossypium hirsutum anthocyanidin 3-O-glucosyltransferase 5 |
| LOC107926612 | XM_016857508 | Gossypium hirsutum anthocyanidin 3-O-glucosyltransferase 5 |
